# The Editorial “We”

**Published:** 1896-04

**Authors:** 


### The Editorial "We."

An exchange says: Somebody who wants to explain what the editorial "we" signifies says the meaning varies to suit the circumstances. For instance, when you read "that we expect our wife home to-day," "we" refers to the editor; when it is "we are a little late with our work," it includes the whole office force, even the devil and towel; "if we are having a boom," the town is meant; "we received over 700,000 immigrants last year," and it embraces the nation, but "we have hog cholera in our midst," only refers to the illness of the man who takes the journal two or three years without paying for it.

